# Effect of Antibiotic Amphotericin B Combinations with Selected 1,3,4-Thiadiazole Derivatives on RPTECs in an In Vitro Model

**DOI:** 10.3390/ijms232315260

**Published:** 2022-12-03

**Authors:** Agnieszka Dróżdż, Adrianna Sławińska-Brych, Dominika Kubera, Magdalena Kimsa-Dudek, Joanna Magdalena Gola, Jolanta Adamska, Celina Kruszniewska-Rajs, Arkadiusz Matwijczuk, Dariusz Karcz, Wojciech Dąbrowski, Andrzej Stepulak, Mariusz Gagoś

**Affiliations:** 1 Department of Cell Biology, Maria Curie-Sklodowska University, Akademicka 19, 20-033 Lublin, Poland; 2 Department of Nutrigenomics and Bromatology, Faculty of Pharmaceutical Sciences in Sosnowiec, Medical University of Silesia, 40-055 Katowice, Poland; 3 Department of Molecular Biology, Faculty of Pharmaceutical Sciences in Sosnowiec, Medical University of Silesia, 40-055 Katowice, Poland; 4 Department of Biophysics, University of Life Sciences, Akademicka 13, 20-950 Lublin, Poland; 5 Department of Chemical Technology and Environmental Analytics, Cracow University of Technology, 31-155 Krakow, Poland; 6 I Clinic of Anaesthesiology and Intensive Therapy with Clinical Paediatric Department, Medical University of Lublin, Jaczewskiego 8, 20-090 Lublin, Poland; 7 Department of Biochemistry and Molecular Biology, Medical University of Lublin, 20-093 Lublin, Poland

**Keywords:** 1,3,4-thiadiazole derivatives, amphotericin B, RPTEC model, caspase activity, cell viability, cell apoptosis, cell necrosis, ROS production, ATR-FTIR spectroscopy

## Abstract

4-(5-methyl-1,3,4-thiadiazole-2-yl) benzene-1,3-diol (C1) and 4-[5-(naphthalen-1-ylmethyl)-1,3,4-thiadiazol-2-yl] benzene1,3-diol (NTBD) are representative derivatives of the thiadiazole group, with a high antimycotic potential and minimal toxicity against normal human fibroblast cells. The present study has proved its ability to synergize with the antifungal activity of AmB. The aim of this work was to evaluate the cytotoxic effects of C1 or NTBD, alone or in combination with AmB, on human renal proximal tubule epithelial cells (RPTECs) in vitro. Cell viability was assessed with the MTT assay. Flow cytometry and spectrofluorimetric techniques were used to assess the type of cell death and production of reactive oxygen species (ROS), respectively. The ELISA assay was performed to measure the caspase-2, -3, and -9 activity. ATR-FTIR spectroscopy was used to evaluate biomolecular changes in RPTECs induced by the tested formulas. The combinations of C1/NTBD and AmB did not exert a strong inhibitory effect on the viability/growth of kidney cells, as evidenced by the negligible changes in the apoptotic/necrotic rate and caspase activity, compared to the control cells. Both NTBD and C1 displayed stronger anti-oxidant activity when combined with AmB. The relatively low nephrotoxicity of the thiadiazole derivative combinations and the protective activity against AmB-induced oxidative stress may indicate their potential use in the therapy of fungal infections.

## 1. Introduction

In recent years, increasing numbers of drug-resistant and multidrug-resistant fungal strains have been isolated in clinical practice. This phenomenon results in difficulties in treatment, especially in the case of infections of multidrug-resistant species [1,2]. Therefore, the development of new antifungal drugs is a highly important challenge. Combinations of selected 1,3,4-thiadiazole derivatives with amphotericin B, which demonstrates strong synergic antifungal interactions, are promising candidates for such formulas [3]. Such a combination enables the therapeutically effective dose of amphotericin and its toxicity to be reduced several times [3,4]. An important step preceding its application in clinical practice is the evaluation of the cytotoxicity of therapeutic formulas in vitro.

Cytotoxicity is caused by adverse actions of chemical and physical factors on cells [5]. To assess the cytotoxicity of different agents, cell viability tests are commonly used [6,7]. One of the most frequently applied viability assays is MTT, evaluating the NADH-dependent enzymatic activity [8,9,10,11]. The MTT assay is based on the ability of only viable cells to reduce and convert the relatively colorless MTT compound into formazan, which is a dark purple and a strongly lipophilic substance. Thus, photometric assessment of the formazan concentration is proportional to the number of viable cells [9]. Another measure of the cytotoxicity of various agents is the occurrence of cell death, including apoptosis and necrosis [7]. Therefore, for the identification of apoptotic cells and discrimination between apoptosis and necrosis, flow cytometry techniques are usually applied to evaluate changes in cell morphology, phosphatidylserine externalization on the cell surface, collapse of mitochondrial transmembrane potential, DNA fragmentation, and evidence of caspase activation [12,13,14,15,16,17,18]. Environmental stress also contributes to an increased production of reactive oxygen species (ROS), causing an imbalance that leads to cell and tissue damage (oxidative stress) [19]. Excessive ROS exposure results in oxidative damage to nucleic acids and elicits proteotoxic stress, which in turn induces apoptosis and cell death [20,21]. Thus, the evaluation of ROS production provides important information on the toxicity of various factors.

Caspases are one of the main components of apoptosis. These enzymes are divided into initiator (-2, -8, -9, -10) and executioner caspases (-3, -6, -7) [22]. Caspases play a role not only in programmed cell death, but also in such processes as cell differentiation, proliferation, neurodegeneration, maintenance of genome stability, and inflammation [22,23]. Inflammatory caspases include caspase-1, -4, -5, and -12 [22]. The initiator caspase-9 is involved in the intrinsic mechanism of apoptosis activation [22]. It cleaves executioner caspase-3 and -7 [22]. Interestingly, caspase-9 is also involved in the autophagy and maintenance of mitochondrial homeostasis [24]. Another initiator, caspase-2, is involved in genomic homeostasis by the stabilization of p53 after DNA damage and the reduction of oxidative stress [23]. Caspase-3 cleaves specific target proteins [25]. However, by interacting with intracellular signaling components, this enzyme is also involved in the regulation of cell proliferation and differentiation [25].

Attenuated total reflection Fourier transformed infrared (ATR-FTIR) spectroscopy is a powerful tool in biomedical research [26,27,28,29,30]. The analysis of IR spectra provides information on the macromolecular composition [31]. FTIR spectroscopy facilitates the evaluation of biomolecular changes occurring within cells that are exposed to different stress factors and the accompanying oxidative stress-related damage, apoptosis, necrosis, etc. [32,33,34,35]. Therefore, this method is gaining increasing interest as a useful instrument for the assessment of the toxicity of new pharmaceuticals in biological systems and the mechanisms of their action, determination of patomechanisms of diseases, or development of spectral markers for medical diagnosis, etc. [4,36,37,38,39,40,41,42,43].

In the present research, we analyzed the effect of two 1,3,4-thiadiazole derivatives, designated as C1 (4-(5-methyl-1,3,4-thiadiazole-2-yl) benzene-1,3-diol) and NTBD (4-[5-(naphthalen-1-ylmethyl)-1,3,4-thiadiazol-2-yl] benzene1,3-diol), and their combinations with AmB in a human renal proximal tubular endothelial cell (RPTEC) line model. The choice of the formulas was dictated by their high synergic antifungal interaction against *Candida* species demonstrated in our previous study (article in press). The RPTEC model was chosen, as renal cells are frequently the damage site due to their central and indispensable role in the filtration of drugs from blood circulation [44,45,46]. The methods applied to evaluate the potential in vitro toxicity of the formulas include the determination of caspase activity in the treated cells and assessment of RPTEC viability, apoptosis, necrosis, and ROS production. ATR-FTIR spectroscopy was used for the semi-quantitative analysis of the biomolecular changes in the cells induced by the compounds and their compositions. 

## 2. Results

### 2.1. Mitochondrial Dehydrogenase Activity

**Figure 1:**
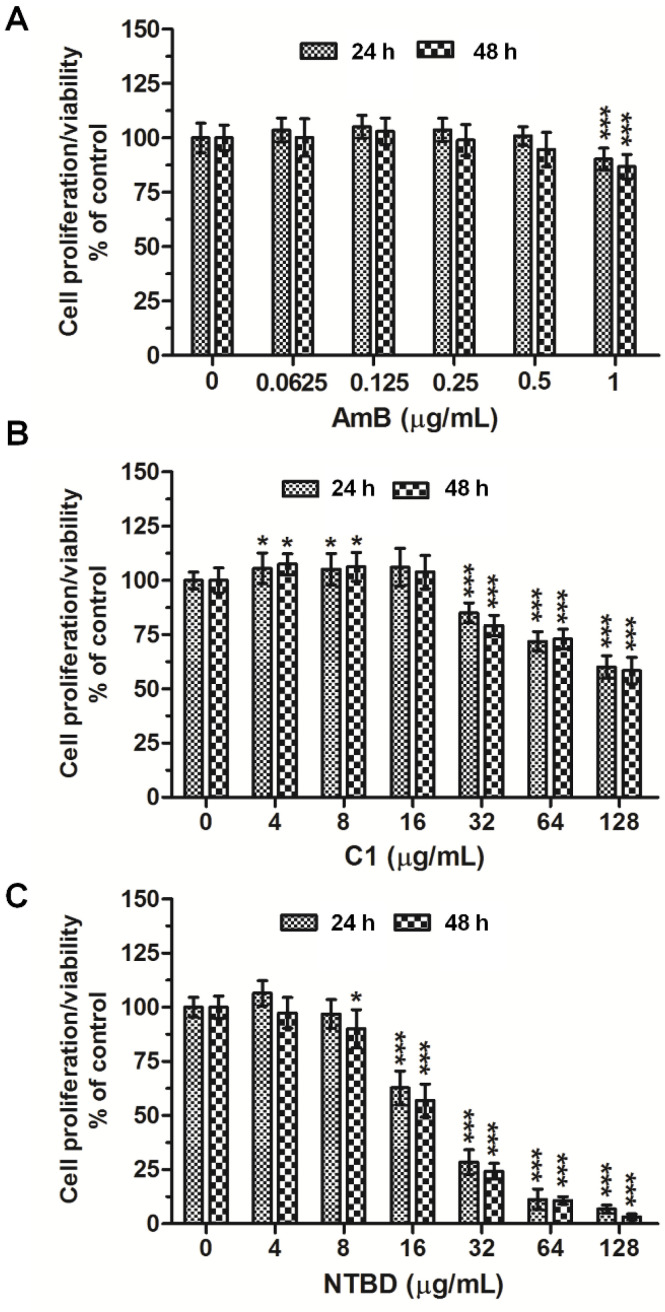
The impact of AmB and thiadiazols C1 and NTBD on the viability of RPTEC cells. Cells were incubated with or without increased concentrations of AmB (**A**), C1 (**B**), or NTBD (**C**) for 24/48 h, and the cell viability/mitochondrial dehydrogenase activity was assessed by MTT assay. Results were expressed as % viability of cells as compared to vehicle (DMSO) control (mean ± SD). Values are significantly different from control, if *p <* 0.05 *, *p <* 0.001 ***, using one-way ANOVA test. Results are shown as a representative of three independent experiments.

Figure 1A–C shows the levels of cytotoxicity of AmB and the two 1,3,4-thiadiazole derivatives C1 and NTBD against normal RPTECs assayed by the MTT test. The results revealed that AmB at concentrations from 0.0625 to 0.5 µg/mL was not harmful to the cells in both 24 h and 48 h cultures. However, a significant suppression of their proliferation occurred at the dose of 1 µg/mL (Figure 1A). The 24 h incubation with C1 in the concentration range of 0–16 µg/mL did not affect the survival of the RPTECs. Only when exposed to higher concentrations (from 32 to 128 µg/mL) did the renal tubular cells show a dose-dependent decrease in mitochondrial dehydrogenase activity. The longer (48 h) exposure did not result in marked potentiation of this compound (Figure 1B). In contrast to C1, NTBD exhibited a stronger toxicity to normal cells. As presented in Figure 1C, the 24 h incubation with this compound caused a statistically significant reduction in cell viability starting from the dose of 16 μg/mL, and even from 8 µg/mL in the 48 h incubation variant.

**Figure 2:**
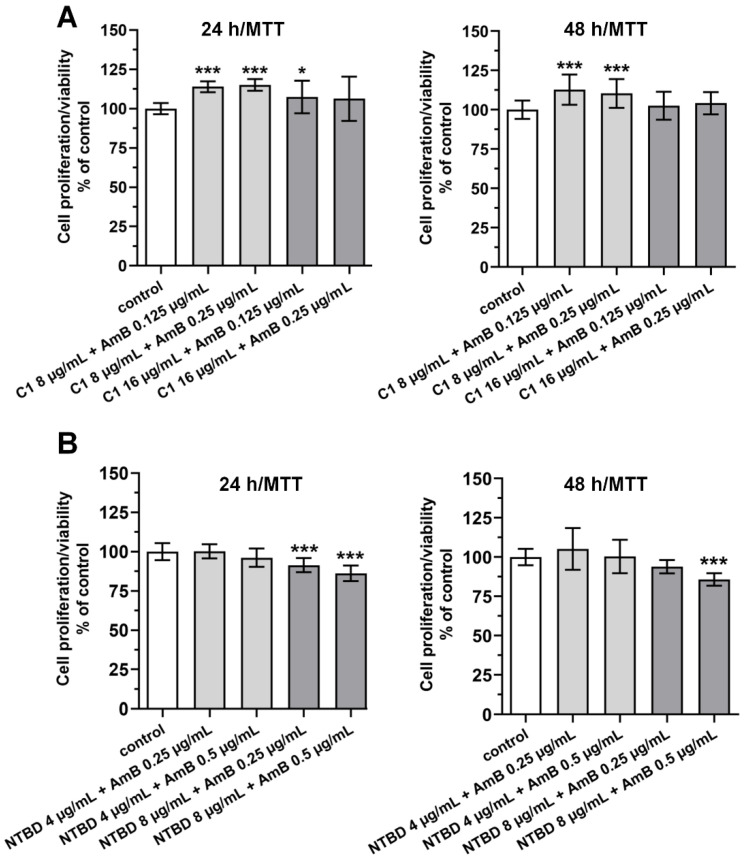
The impact of the combination of AmB with thiadiazols C1 (**A**) or NTBD (**B**) on the viability of RPTEC cells. The cells were incubated with either AmB, C1, and NTBD alone or with the mixture AmB + C1 (**A**) and AmB + NTBD for 24/48 h, and the cell viability/mitochondrial dehydrogenase activity was assessed by MTT assay. Results were expressed as % viability of cells as compared to vehicle (DMSO) control (mean ± SD). Values are significantly different from control if *p <* 0.05 *, *p <* 0.001 ***, using the one-way ANOVA test. Results are shown as a representative of three independent experiments.

Figure 2 shows the cytotoxicity against RPTECs cultured in the presence of the combination of C1 or NTBD with AmB. The addition of C1 (either 8 or 16 µg/mL) to AmB (0.125 or 0.25 µg/mL) did not disturb the proliferation/viability of the RPTECs within 24 h, compared to the vehicle control (Figure 2A; upper left panel). Moreover, the 2-day treatment of the RPTECs with the mixture of C1 and AmB had no inhibitory effect on their metabolic activity (in each of the drug combinations used, the cell viability was over 100%, Figure 2A; upper right panel). In the 24/48 h combined NTBD + AmB treatment, a pronounced decrease in cell viability was only observed at 8 µg/mL of NTBD. In turn, the use of the lower dose of NTBD in combination with AmB had a stimulatory (although non-statistical) impact on the growth of the RPTECs (Figure 2B).

### 2.2. Impact of Thiadiazole Derivatives and Their Combinations with AmB on Necrotic and Apoptotic Cell Death in RPTEC Culture

**Figure 3:**
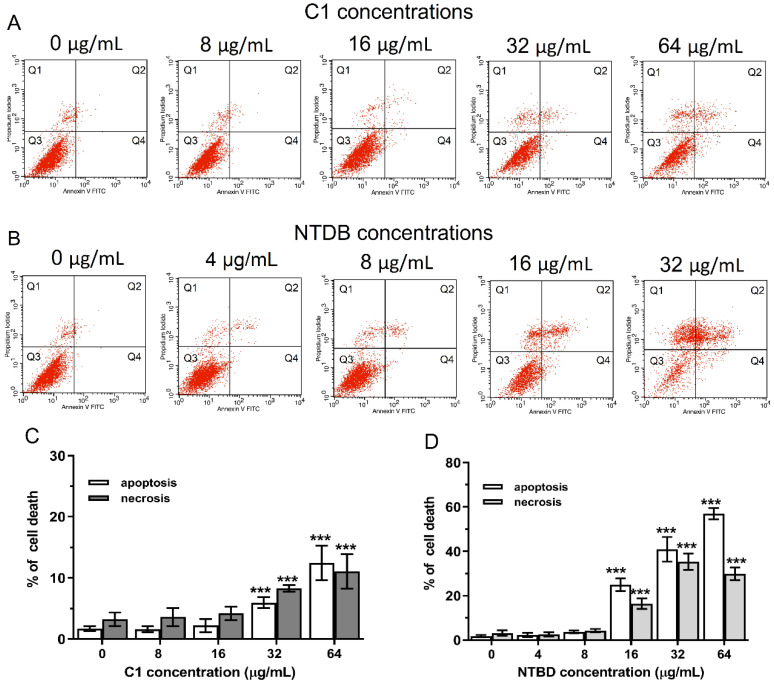
Impact of C1 and NTBD on the induction of apoptotic and necrotic cell death in the RPTEC cells. Following the 24 h incubation with or without the increasing concentrations of C1 or NTBD, the cells were double stained with Annexin-V/PI and analyzed with flow cytometry. Histograms (**A**,**B**) show the population of viable and dead cells, and quantitative analyses of the percentage of apoptotic (early + late apoptosis) and necrotic cells are presented in the form of bar graphs (**C**,**D**), for C1 and NTBD, respectively. Each red dot on the dot plots indicate single event/cell. Values are significantly different from the vehicle control if *p <* 0.001 *** in the one-way ANOVA test. Results are shown as a representative of three independent experiments. Abbreviations: Q1—An-/PI+ necrotic cells, Q2—An+/PI+ late apoptotic cells, Q3—An-/PI- viable cells, Q4—An+/PI- early apoptotic cells.

**Figure 4:**
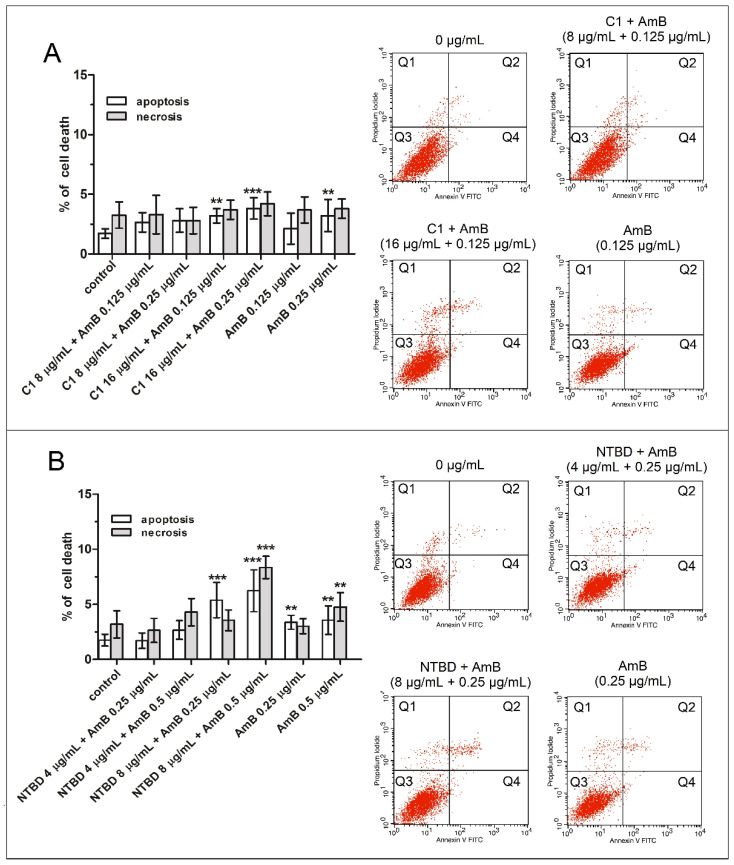
Impact of the combinations of AmB with thiadiazoles C1 (**A**) or NTBD (**B**) on the induction of apoptotic and necrotic cell death in RPTECs. Following the 24 h treatment with or without a mixture of AmB with C1 or NTBD and with each of these compounds alone, the cells were double stained with Annexin-V/PI and analyzed with flow cytometry. Each red dot on the dot plots indicate single event/cell. Bar graphs show quantitative analyses of the percentage of apoptotic (early + late apoptosis) and necrotic cells. Values are significantly different from the vehicle control if *p <* 0.01 **, *p <* 0.001 *** in the one-way ANOVA test. Selected histograms from three independent cytometric measurements are presented on the right side of the graphs. Abbreviations: Q1—An-/PI+ necrotic cells, Q2—An+/PI+ late apoptotic cells, Q3—An-/PI- viable cells, Q4—An+/PI- early apoptotic cells.

The 24 h treatment with C1 and NTBD alone resulted in a dose-dependent elevation in the apoptosis and necrosis levels (Figure 3A–D). In the case of C1, a statistically significant increase in both populations of dead cells was observed at concentrations of 32 and 64 µg/mL (Figure 3A,C), whereas a clear induction of cell death was noted at 16 µg/mL of NTBD (Figure 3B,D). In turn, the use of an amphotericin dose of 0.5 µg/mL resulted in the greatest increase in the number of destroyed cells (including apoptotic and necrotic cells), compared to the control. In contrast, the concentration of 0.25 µg/mL induced slight changes, but only in the fraction of apoptotic cells (Figure 4).

In the 24-h combined treatment, the administration of AmB (0.125 or 0.25 µg/mL) with 8 µg/mL C1 elicited a negligible proapoptotic response in the RPTEC cell cultures. Moreover, at the higher doses of C1 (16 µg/mL) with AmB, there was no rapid increase in apoptosis, which reached a maximum value of 3.8%. However, compared to the AmB alone treatment, the percentage of apoptosis was slightly higher. Importantly, none of the combinations applied triggered a significant increase in the necrosis rate (Figure 4A). Similarly, the combination of AmB (0.25 or 0.5 µg/mL) with a low dose of NTBD (4 µg/mL) had no relevant effect on promoting either necrosis or apoptosis, compared to the control cells. However, when the cells were treated with the mixture of 0.5 µg/mL AmB and 8 µg/mL NTBD, there was an approximately three-fold increase in the number of apoptotic and necrotic cells in relation to the untreated group (Figure 4B).

### 2.3. Impact of Thiadiazole Derivatives and Their Combinations with AmB on Intracelluar ROS Production

**Figure 5:**
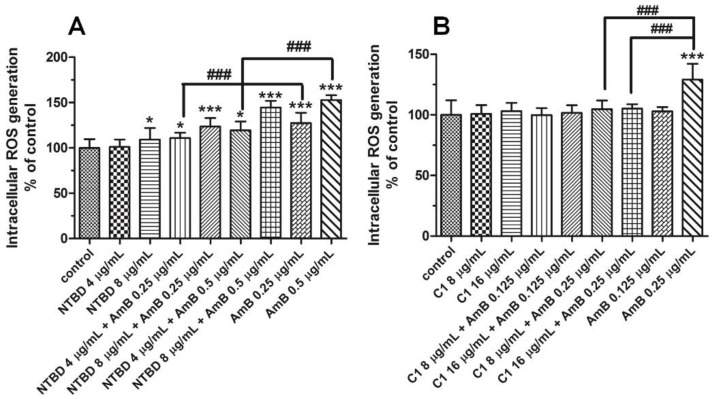
The impact of the combination of AmB with thiadiazols NTBD (**A**) or C1 (**B**) on ROS production in RPTEC cells. Following the 12 h treatment with or without a mixture of AmB with C1 or NTBD, and with each of these compounds alone, the ROS content was evaluated using dihydrorhodamine-123 probe as described in Method section. Data are shown as means S.D. of three independent experiments and analyzed by one-way ANOVA, followed by Dunnett’s multiple comparisons test. (*p* < 0.05 *, *p* < 0.001 *** vs. control, ### *p* < 0.001 vs. AmB).

The experiments with DHR 123 staining showed that, of the two thiadiazoles tested, only the NTBD molecule (after 12 h) exhibited pro-oxidant activity, with a statistically significant difference from the control group observed at the concentration of 8 µg/mL (Figure 5A). The ability to produce oxygen free radicals was also manifested by amphotericin B at the dose of 0.25 and higher (Figure 5A,B). Interestingly, the administration of C1 at concentrations of 8 or 16 µg/mL, together with 0.25 µg/mL AmB, clearly counteracted the AmB-induced ROS generation (Figure 5B). In addition, the co-treatment with NTBD and AmB was found to have a dose-drug dependent impact on ROS production, compared to the control, and the highest levels of ROS were noted in the variant with the mixture of 8 µg/mL NTBD + 0.5 µg/mL AmB. Nevertheless, the combined effect of these compounds (especially with the lowest dose of NTBD) caused much less oxidative stress than that triggered by AmB used alone. As indicated in Figure 5A, the combination of 0.5 µg/mL AmB with 4 µg/mL of NTBD reduced the ROS level by approximately 33%, compared to the cells treated with 0.5 µg/mL AmB.

Based on the results presented in Section 2.1, Section 2.2 and Section 2.3, and our previous studies on the synergic antifungal interactions of 1,3,4-thiadiazole derivatives, the following combination were chosen for further analysis: C1 (8 µg/mL) + AmB (0.125 µg/mL) and NTBD (4 µg/mL) + AmB (0.25 µg/mL).

### 2.4. Effect of Thiadiazole Derivatives and Their Combinations with AmB on the Activity of Caspases in RPTEC Culture

**Figure 6:**
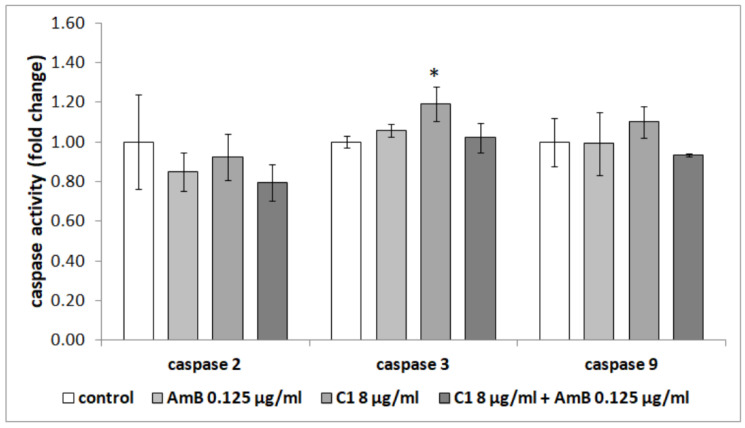
Effect of the combination of AmB with thiadiazole C1 on the activity of caspases in the RPTEC cells. The results are presented as fold changes relative to the control cells and are reported as the mean ± SD, statistical significance: * *p* < 0.05 (Tukey post hoc test).

The analysis of the effect of thiadiazole derivatives and their combinations with AmB on the RPTECs also consisted of the determination of caspase activity in these cells, both initiator (caspases-2 and -9) and effector caspases (caspase-3). It was found that only the activity of effector caspase-3 was slightly higher in the cells treated with C1 at the concentration of 8 µg/mL after 24 h, compared to the control cells treated with 0.125 µg/mL of AmB, and cells treated with the combination of these compounds (*p* < 0.001, *p* = 0.012 and *p* = 0.002, respectively, Tukey post hoc test). However, C1 (8 µg/mL) in combination with AmB at a concentration of 0.125 µg/mL did not affect the activity of any of the tested caspases (Figure 6). Similarly, caspase activity was not affected by a lower concentration of AmB—0.125 µg/mL.

**Figure 7:**
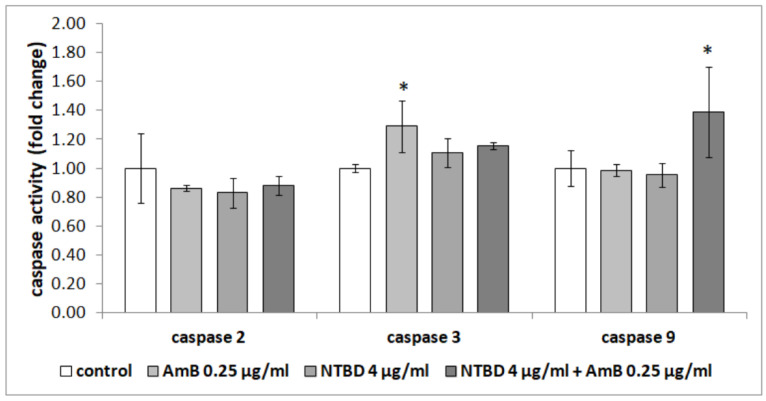
Effect of the combination of AmB with thiadiazole NTBD on the activity of caspases in the RPTECs. The results are presented as fold changes relative to the control cells and are reported as the mean ± SD, statistical significance: * *p* < 0.05 (Tukey post hoc test).

It was also observed that amphotericin at the concentration of 0.25 µg/mL caused a significant increase in the activity of effector caspase-3 compared to the untreated cells (*p* = 0.005, Tukey post hoc test). In combination with NTBD (4 µg/mL), it increased the activity of initiator caspase-9, compared to all the studied groups (*p* = 0.040 vs. control, *p* = 0.033 vs. AmB 0.25 µg/mL and *p* = 0.020 vs. NTBD 4 µg/mL, Tukey post hoc test) (Figure 7). 

### 2.5. Semi-Quantitative ATR-FTIR Analysis of the C1+AmB and NTBD+AmB In Vitro Effect in the RPTEC Model

**Figure 8:**
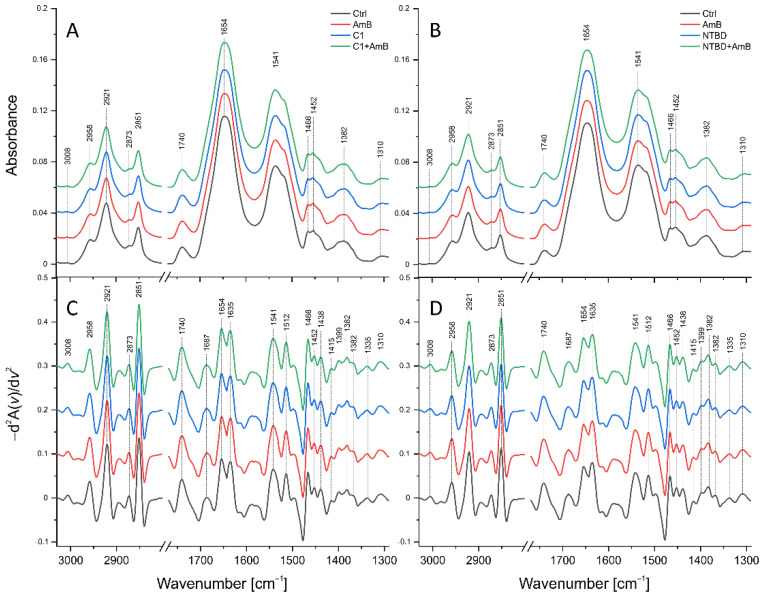
Mean ATR-FTIR spectra of RPTEC cells: (**A**)—control, treated with AmB (0.03 μg/mL), C1 (8 μg/mL), and their mixture; (**C**)—reversed second derivatives of spectra presented in (**A**); (**B**)—control, treated with AmB (0.06 μg/mL), NTBD (4 μg/mL), and their mixture; (**D**)—reversed second derivatives of spectra presented in (**B**).

The mean ATR-FTIR spectra of the control RPETC cells and those treated with C1 (8 µg/mL), AmB (0.125 µg/mL), and their mixture are presented in Figure 8A, while the mean IR spectra of RPTECs subjected to NTBD (4 µg/mL), AmB (0.25 µg/mL), their mixture, and the corresponding control are shown in Figure 8B. In order to the enhance spectral differences and resolve the problem of the overlapping components in the IR absorption bands, reversed second derivatives of the aforementioned spectra were calculated and presented in Figure 8a,b, respectively. Based on the reversed second derivatives, the spectral assignments were performed, as presented in Table 1.

**Figure 9:**
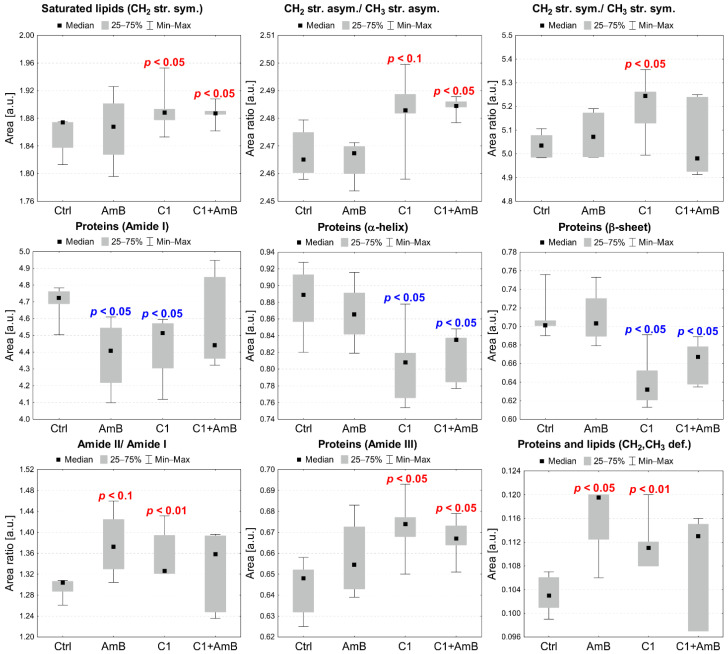
Median, minimal, and maximal values of the intensities of selected absorption bands, presenting differences between RPTEC subjected to the C1, AmB, and C1 + AmB treatment and the control (Ctrl) group. The *p*-values of the Mann–Whitney U test for statistically significant changes, compared to the control, are shown in the charts (red—increases, blue—decreases).

**Figure 10:**
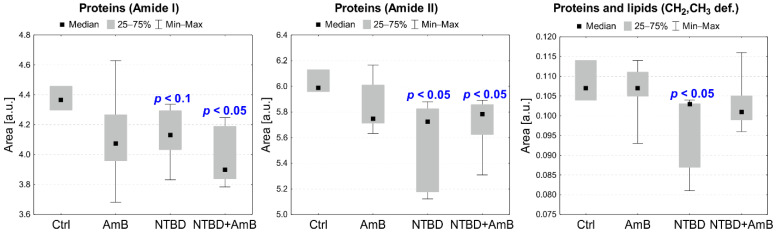
Median, minimal, and maximal values of intensities of selected absorption bands presenting differences between RPTEC subjected to the NTBD, AmB, and NTBD+AmB treatment and the control (Ctrl) group. The *p*-values of the Mann–Whitney U test for statistically significant changes, compared to the control, are shown in the charts (red—increases, blue—decreases).

The differences in the identified band intensities in the spectra of RPTECs treated with C1, AmB, and their mixture as well as NTBD, AmB, and their combination were analyzed based on the integral areas of peaks calculated based on the reversed second derivatives of the IR spectra and compared to the control. The statistical significance of the observed changes was assessed with the Mann–Whitney U test at a significance level of 5%. Additionally, statistical trends were analyzed at a significance level of 10%. The results obtained for selected absorption bands or band ratios are presented in Figure 9 and Figure 10.

As presented in Figure 9, the analysis revealed a statistically significant increase in the intensity of the absorption band of CH_2_ symmetric stretching vibrations (2921 cm^−1^) and an elevated ratio of CH_2_ and CH_3_ asymmetric stretching vibrations (2921 cm^−1^/2958 cm^−1^) in the RPTECs treated with C1 and C1+AmB. In the C1-treated RPTECs, the relative content of CH_2_ and CH_3_ symmetric stretching vibrations (2851 cm^−1^/2873 cm^−1^) was also found to be significantly higher than in the control. The overall Amide I (maximum at 1654 cm^−1^) content was relevantly diminished in the cells treated with AmB and C1; however, it did not change in the RPTECs subjected to C1+AmB in relation to the control. The α-helix (1654 cm^−1^) and β-sheet (1635 cm^−1^) levels did not vary in the AmB-treated cells but were significantly lower in the C1 and C1+AmB variant, compared to the control group. In the RPTECs subjected to AmB and C1, an elevated amide II to amide I ratio (1641 cm^−1^/1654 cm^−1^) and an increased level of CH_2_ and CH_3_ deforming vibrations (1360–1460 cm^−1^) related to the protein and lipid content were found. Finally, the amide III (maxima at 1310 cm^−1^ and 1335 cm^−1^) level was significantly higher in the AmB- and C1-treated cells, compared to the control.

Figure 10 presents statistically significant changes in the biomolecular content of RPTECs treated with AmB, NTBD, and their mixture. In the cells subjected to NTBD and NTBD + AmB, a statistically significant decrease was observed in Amide I (1654 cm^−1^) and Amide II (1641 cm^−1^), compared to the control. A reduced level of absorption bands originating from CH_2_ and CH_3_ deforming vibrations (1360–1460 cm^−1^) was also found but only in the NTBD-treated RPTECs.

## 3. Discussion

Amphotericin (AmB, a macrocyclic polyene antibiotic) is the first-line drug used for the treatment of severe invasive fungal infections. The selective ability of this drug to bind to ergosterol, i.e., a key component of fungal cell membranes, determines its high fungistatic and fungicidal potential. Although its affinity for ergosterol is much stronger than that for cholesterol (the main animal sterol), AmB exerts a certain effect on mammalian cells [55,56]. Due to the high content of this sterol in the membranes of renal tubular cells, the kidneys are particularly sensitive to its action [57]. The nephrotoxicity of amphotericin B is often manifested by hypokalemia, hypomagnesemia, uremia, metabolic acidosis, and, less frequently, acute tubular necrosis [56,58,59]. The continuous increase in the incidence of AmB-induced acute or chronic kidney injury indicates an urgent need to modify the current antifungal treatment regimens and/or develop new potential solutions [60,61,62,63,64]. 

In this context, a combination therapy based on the administration of AmB (in sub-toxic doses) simultaneously with other non-toxic medicinal substances that have been proved to enhance the therapeutic effects of the antibiotic seems to be one of the promising treatment options. The use of two or more pharmaceuticals with different mechanisms of action often yields better clinical results than monotherapy. Hence, the multiyear research conducted by our team has focused on the search for new biomolecules and their interactions with AmB. Recent studies, conducted by Chudzik [3,4], have indicated 1,3,4-thiadiazole derivatives as a group of synthetic compounds with potential antimycotic activity. The research results published in PLOS suggest that the compound 4- (5-methyl-1,3,4-thiadiazole-2-yl) benzene-1,3-diol, abbreviated as C1, is one of the most active thiadiazoles against fungal pathogens. In in vitro studies, it was shown to inhibit the growth of various *Candida* species, including azole-resistant isolates and molds, with MIC100 values ranging from 8 to 96 μg/mL. Its minimal toxicity to normal cells should be emphasized. This derivative used alone and in combination with AmB (in low and moderate doses) did not affect the metabolic activity of the human skin fibroblast NHDF line [4]. Only one compound of the large group of newly synthesized 1,3,4 thiadiazole derivatives studied recently, i.e., NTBD, has been found to be effective in synergizing the fungicidal properties of AmB (article in press). Despite the demonstrated therapeutic activity of C1 and NTBD and their beneficial interaction with amphotericin B, the effect of these compounds and their combinations with AmB on renal cell function is still unclear. To clarify this issue, we used a commercially available primary culture of human renal proximal tubule epithelial cells (RPTECs). This line is not genetically modified; hence, it is an excellent tool for in vitro studies of the mechanisms of action of various drugs. To assess the cytotoxicity of these thiadiazoles and their combinations with AmB, we chose techniques for the analyses of mitochondrial cell metabolism and induction of cell death and oxidative stress.

The preliminary results obtained with the use of the MTT method indicated a varied cytotoxicity of the tested thiadiazole derivatives against the RPTECs. The lowest doses of NTBD and C1 inducing disturbances in the metabolic activity after 24–48 h were 8 µg/mL and 32 µg/mL, respectively. The effects of these compounds were enhanced by the further increases in their concentrations. At the highest dose (128 µg/mL), the cell viability/proliferation decreased by almost 43% (C1) and as much as 97% (NTBD). AmB was the most potent compound of all the substances tested, as it caused a statistically significant reduction in the number of metabolically active cells even at the very low dose of 1 µg/mL. These results confirm previous reports on the negative impact of higher AmB doses on the vital parameters of renal cells and are consistent with findings shown by other authors postulating the low toxicity of various 1,3,4-thiadiazole derivatives to healthy human and animal cells [65,66,67,68,69]. Based on the present and previous observations, it can be concluded that the high antifungal potential of C1 is not related to its toxicity, because neither the human skin fibroblasts (NHDF) nor the RPTECs (two morphologically and physiologically different cell types) responded to its wide concentration range. Model analyses on liposomes carried out with the use of 1,2-dipalmitoyl-sn-glycero-3-phosphatidylcholine (DPPC) [70] may partially explain the cause of the low toxicity of C1 against human cell cultures. As suggested by the authors, the limited ability of C1 to interact with the hydrophobic part of membrane phospholipids, with the simultaneous stronger affinity for hydrophilic groups present in the cell membrane, may hinder the free penetration of this molecule into the cell. The question of the lower tolerance of the tested cells to the other derivative still must be clarified. The higher biological activity of NTBD may be related to the presence of a molecular target specific to this molecule. As proved by Matwijczuk et al. (2015) [71], this compound may exist in two tautomeric forms, i.e., the enol and ketone forms, which interact differently with biological systems. The process of intramolecular proton transfer often changes the chemical nature of the compound and the pharmacological properties of the molecule. Hence, the analyzed cells may have reacted more strongly to this thiadiazole. More extensive research is necessary to confirm our assumptions. 

In the subsequent MTT analyses, assessing the effects of the combined action of AmB with the thiadiazoles (used at low, non-toxic concentrations), we showed that only the combination with NTBD had some inhibitory effect on the mitochondrial oxidoreductive potential in the renal tubular cells. The highest approx. 15% decrease in the number of metabolizing cells was recorded after the simultaneous incubation of the cells with 8 µg/mL NTBD and 0.5 µg/mL AmB. In the case of the other derivative (C1), there were no tangible changes in the absorbance values in any of its combinations with AmB, which proves the absence of antiproliferative/cytotoxic activity of these complexes. Similar observations were reported by Chudzik et al. (2019) [3]. The present findings allow a conclusion that the analyzed drug compositions, especially those with C1, are characterized by a high safety profile.

To analyze the results of the MTT-based cytotoxicity tests, we made parallel cytometric measurements of the control and experimental cells, stained with annexin V and propidium iodide. The flow cytometry data indicate that, depending on the dose, the derivatives may already interfere with signaling pathways associated with genetically programmed cell death (apoptosis) after 24 h. It was found that NTBD only exhibited no toxicity at the first two doses (4 and 8 µg/mL). In the case of the less toxic C1 compound, the 16 µg/mL dose did not exert a significant effect either. In turn, both compounds applied in the higher concentration range (32–64 µg/mL) significantly increased the population of apoptotic cells. The highest percentage of such cells was recorded after the induction with 64 µg/mL of NTBD. In addition to apoptosis, the analyzed compounds exerted different effects on cell membrane permeability and necrotic death. The incubation with the highest dose of C1 and NTBD resulted in a nearly 8% and 26% increase in the number of necrotic cells, respectively, compared to the control. These results confirmed the preliminary MTT results of the toxicity of the higher doses of the thiadiazoles and indicated the involvement of apoptosis and necrosis in the mechanisms of the cytotoxic action of these compounds.

The ability of thiadiazoles to destroy cells was most often analyzed to assess their anti-tumor activity [68,69]. Both in vitro and in vivo model studies have proved the high apoptosis-inducing activity of many members of this class of compounds in various types of neoplastic cells, e.g., breast cancer, lung cancer, colorectal cancer, and blood cancers. Parallel studies on normal cells determined the antiproliferative properties of thiadiazoles. As shown by literature reports, most of these compounds were characterized by good tolerance and low toxicity. This may indicate a selective mode of their action [68,69,72,73,74]. 

The biocidal activity of 1,3,4 thiadiazoles against pathogenic fungi has also been assessed [3,4,75]. Studies on *C. albicans* yeast cell cultures demonstrated a stimulating effect of C1 on necrosis and autophagy induction, but no apoptogenic effect was observed [3,4]. The type of thiadiazole-induced death seems to be largely dependent on the type and origin of the eukaryotic cells. The different expression and number of molecules involved in the cell death signaling pathways in healthy, pathological (neoplastic), or fungal cells, combined with the varied adaptive response and defense of these cells to stress factors, may be responsible for the different mechanisms of action of these compounds in various biological systems [76,77].

Since neither of the analyzed thiadiazoles applied in the low doses had a significant modulating effect on the processes related to apoptotic and necrotic cell death, the equally weak effects of these compounds on these phenomena could be expected in the combined treatment of the cells with AmB. As shown in Figure 3, the C1+AmB combination added to the RPTEC cultures did not result in any significant increase in the number of dead cells, compared to the control group. Similarly, the survival rate of the studied population treated with the highest concentration of C1 (16 µg/mL) was high (approx. 90%). More pronounced changes in the number of apoptotic and necrotic cells were observed only after the administration of AmB (0.5 µg/mL), combined with the other derivative NTBD (8 µg/mL). Moreover, the combined effect of these drugs was almost twice as strong as that of the administration of amphotericin alone (0.5 µg/mL). The results presented above largely coincide with the spectrophotometric data (Figure 2) and suggest that the low cytotoxicity of these complexes was an effect of the induction of cell death rather than the inhibition of cell divisions.

Although apoptosis is regarded as a physiological form of death, its intensity in many cases has pathogenic effects. Renal tubules are kidney structures with the highest susceptibility to the action of toxic agents. In the case of damage to these structures, the kidney triggers repair processes, including those related to cell proliferation and remodeling. However, a large loss of cell mass as a result of the excessive activation of pro-apoptotic signals may impair the effective remodeling and repair of renal tissue at the damage sites. It is worth mentioning that mass apoptosis promotes cell infiltration and the activation of fibrogenic cells, which is related to the fibrotic process. Hence, besides necrosis, apoptosis is one of the elements of the pathophysiology of renal diseases [78]. 

The literature data increasingly highlight the role of apoptosis in structural and functional kidney disorders caused by amphotericin B [62,66,67,79]. The ability of this antibiotic to promote apoptosis and necrosis has been confirmed in a number of renal cell lines. Highly metabolically active proximal tubule cells have been reported to be the most susceptible to pro-apoptotic effects, whereas the lowest susceptibility has been found in the case of distal tubule cells. The dose-dependent and renal cell origin-dependent pro-apoptogenic potential of AmB has been demonstrated in experiments on animal models [67]. The correlation between the intensity of apoptosis and the clinical symptoms of AmB-induced nephrotoxicity, as well as the reduction in the number of side effects caused by the anti-apoptotic factor rhIGF-1, provide direct evidence for the involvement of apoptosis in the mechanisms of nephrotoxicity of this antibiotic. Thus, a combination of therapies capable of blocking multiple regulated cell death pathways might ensure cell survival and renal function.

In the present study, the RPTECs were incubated with low doses of AmB; hence, no significant induction of apoptosis was achieved. In line with the findings reported by other authors, only the 1 µg/mL dose had a significant impact on the dynamics of this process [67]. Since the combined therapy with AmB and the thiadiazoles only weakly sensitized the primary renal cells to apoptosis, it can be expected that the in vivo interaction of these compounds will not increase the risk of damage to proximal tubular epithelial cells.

In addition to changes in the cell membrane structure consisting in phosphatidylserine (PS) externalization, the induction of caspases is another biochemical marker of apoptosis [80]. The activity of these enzymes is associated with both cell death and the inflammation response, and the caspase cascade can be initiated by various factors [81]. Moreover, the dysregulation of caspase functions may lead to many pathological conditions. Fu et al., (2011) showed that amphotericin B induced apoptosis in *Candida albicans* through an increase in the expression of the *CaMCA1* gene-encoding metacaspase and in caspase activity. This effect can be enhanced by various compounds [82,83]. In turn, Varlam et al., (2001) confirmed the pivotal role of caspase-3 in apoptosis, induced by AmB in a dose-dependent manner in renal tissue culture cell lines [67]. Hence, our study was also concentrated on the caspase activity in human cells after treatment with AmB or AmB, in combination with thiadiazole derivatives, to confirm their effect on the RPTECs. Importantly, we indicated that the selected concentrations of these compounds did not have a significant effect on caspase activity, and consequently, the induction of RPTEC apoptosis. Furthermore, Mostafa et al., (2022) demonstrated that some thiadiazole derivatives had anti-apoptotic properties in the kidneys of rats through caspase inhibition [84].

Human cells exposed to various cytotoxic substances often experience oxidative stress related to a disturbance in homeostasis and the overproduction of oxygen free radicals (ROS). At physiological concentrations, reactive oxygen species do not pose a threat to the organism. These molecules mediate the regulation of metabolism and the vital functions of the cell by interfering with the signaling pathways related to cell proliferation, survival, and differentiation. However, at high concentrations, they exert many adverse effects (e.g., damage to macromolecules, an increase in the cytoplasmic concentration of calcium ions, enhancement of cell membrane permeability, inhibition of ATP production, or a decrease in metabolic activity), contributing to destruction of tissues and apoptotic cell death [85,86,87]. 

Many authors have reported that AmB nephrotoxicity is associated with intracellular oxidative damage and a pro-inflammatory effect [66]. The mechanisms of AmB-induced oxidative stress are still poorly understood. They include lipid peroxidation with the generation of oxygen free radicals, modulation of redox enzyme activity, or electron leakage in the mitochondrial respiratory chain [58,88,89,90]. Previous research carried out by our team has evidenced the pro-oxidative activity of AmB, e.g., in *C. albicans* and *C. parapsilosis* cells and in cultures of human skin fibroblasts and tubular epithelial cells [3,65]. Similarly, the present experiments showed an increase in the intracellular accumulation of ROS caused by AmB, but this effect was observed at much lower concentrations. Our results suggest that AmB can disturb the cellular redox balance even in minimal doses. Taking into account the antioxidant activity of the many synthetic representatives of the 1,3,4-thiadiazole group [87,91], the subsequent step of the study consisted of analyses of the impact of the compounds and their combination with AmB on the oxidative status in the RPTEC cells. As shown in Figure 5, both thiadiazoles had the ability to neutralize free radicals, but only in the presence of AmB. In contrast, neither of the compounds exerted an antioxidant effect when used alone. The comparison of the protective action of both thiadiazoles against the ROS-stimulating action of AmB revealed greater effectiveness of C1. In the combinations with NTBD, a significant decline in the amount of oxygen free radicals (compared to the AmB-treated group) was noted only in the variants with the lowest dose of the compound.

Since the pro-oxidative effect of amphotericin B plays an important role in modulating the immune response in patients treated with this antibiotic and contributes to the overproduction of pro-inflammatory mediators and the intensification of inflammation in the endothelium and renal interstitial tissue [92], the ability of the analyzed derivatives to inhibit endogenous ROS synthesis seems to be an additional advantage of these compounds. The beneficial effects of the thiadiazoles shown in the present study may protect renal cells against the cytotoxic effects of oxygen free radicals, including apoptosis. The available experimental data suggest that the upregulation of the *SOD2* gene encoding mitochondrial superoxide peroxidase 2, which is the first line of defense against peroxides generated via oxygen phosphorylation, is one of the defense mechanisms of RPTECs against the toxic effects of AmB [65]. The expression of this enzyme may be enhanced in the presence of thiadiazoles, which may explain the cause of the decrease in the level of these harmful molecules. It cannot be ruled out that other antioxidant mechanisms related to these derivatives are involved in the cell defense against drug-induced oxidative stress. Further studies are required to clarify this issue.

FTIR-ATR spectroscopy was applied to investigate the semi-quantitatively bimolecular changes induced by 1,3,4-thiadiazole derivatives, AmB, and their combinations in the RPTEC in vitro model after the 24-h exposure. A set of spectral parameters, including the absolute and relative levels of the selected IR absorption bands, were analyzed. The study of changes in the absolute intensities of the bands provides information about fluctuations in the content of the corresponding biomolecules, i.e., lipids and proteins, while the analysis of appropriate band ratios may indicate abnormalities in the molecular structure [31,36,37]. In the analyzed groups, more biomolecular changes were found in the RPTECs treated with C1 and C1+AmB than in those subjected to NTBD and NTBD+AmB. In the current study, the increase in the absolute level of saturated lipids and in the CH_2_/CH_3_ asymmetric stretching ratio was observed for the RPTECs treated with C1 and C1+AmB, while no significant anomalies in the lipid-related spectral parameters were found in cells subjected to AmB, NTBD, and NTBD+AmB. The changes in the relative content of the CH_2_ and CH_3_ groups may be related to alterations in the lipid chain length, branching, and/or saturation level induced by oxidative stress [36,54]. The oxidative stress-related cell damage also results in changes in the protein secondary structure, manifested by an elevated relative β-sheet to α-helix ratio in IR spectra [32,36,37,93]. Despite the decrease in the level of both secondary protein structures in the RPTECs treated with C1 and C1+AmB, no anomalies in their relative abundance were found in any of the analyzed groups. The increase in the CH_2_ symmetric stretching absolute level and the abundance of CH_2_ and CH_3_ deforming vibrations is regarded as an apoptotic marker connected with phosphatidylserine (PS) conformational changes accompanying its externalization in cells. Yet, as reported in the literature, in addition to the changes in the CH_2_ and CH_3_ levels, enhancement of the intensity of the C=O stretching vibration band also occurs during PS apoptotic activation [34,35,40]. Therefore, as no simultaneous increase in CH_2_ asymmetric stretching nor C=O stretching absorption bands was found, we can assume that the observed anomalies are not associated with PS apoptotic activity. However, the observed changes in the amide I level in the RPTECs treated with AmB and C1, in particular the distortion in the relative level of amide II and amide I, may indicate the activation of such apoptotic proteins as caspase-3 [40]. Changes in the absolute amide I and amide II level were observed in the RPTECs subjected to NTBD and NTBD+AmB; however, there were no abnormalities in the amide II and I relative abundance in these groups. In the case of RPTECs treated with C1 and C1+AmB, the reversed behaviors of the amide I and III proteins were observed. This may be explained by the fact that the amide III absorption region is overlapped by the CH_2_ wagging vibrations of lipids [31]. This seems to be supported by the observed correlation between the levels of amide III and saturated lipids (CH_2_ str. sym.). It should be highlighted that the ATR-FTIR semi-quantitative analyses were performed in the variant with the 24-h exposure of the RPTECs to the analyzed compounds. However, since the previously discussed results of the cytotoxicity of the compounds and their mixtures showed that the doses subjected to the ATR-FITR semi-quantitative analyses did not cause a significant toxic effect after the 48-h exposure, it may be assumed that the observed biomolecular anomalies are temporary.

Together, with their high antifungal activity, the low toxicity of the AmB complexes with thiadiazoles (C1 and NBTB) towards the renal tubular epithelial cells shown in the study indicates the possibility of a practical use of these combinations to fight pathogenic fungi. However, further in vivo studies are necessary for the final verification of the pharmacological safety profile of the drug compositions and the assessment of their therapeutic potential.

## 4. Materials and Methods

### 4.1. Human Renal Proximal Tubule Cell Culture

Cryopreserved and freshly isolated human renal proximal tubule cells (RPTECs) were purchased from the Lonza Group AG (Lonza Group AG, Basel, Switzerland). The cells were grown for a maximum of four passages in epithelial basal medium (REBM) supplemented with a growth factor cocktail (Lonza catalog no. CC-3190) and gentamycin 100 μg/mL (Sigma-Aldrich Chemical Co., St. Louis, MO, USA). The cells were maintained in an incubator with a humidified atmosphere of 95% air and 5% CO_2_ at 37 °C.

### 4.2. Antifungal Substances

The amphotericin B powder from *Streptomyces* sp. was purchased from Sigma-Aldrich (cat. No. A4888). Its purity (HPLC) was approximately 80%, which was taken into account in the calculations of the concentrations. The 1,3,4-thiadiazole derivatives C1 and NTBD were synthesized in the Department of Chemical Technology and Environmental Analytics at the Cracow University of Technology.

### 4.3. Determination of Mitochondrial Dehydrogenase Activity (MTT Assay)

The cytotoxicity assay was performed by the colorimetric determination of formazan dissolved in the SDS buffer. Due to the mitochondrial dehydrogenase activity, living cells are capable of reducing water-soluble yellow MTT to insoluble purple formazan crystals. Their amount is proportional to the number of metabolically active cells. The RPTECs were trypsinized, seeded at a density of 7 × 10^3^ cells/mL in a volume of 100 μL per well in a 96-well plate (Thermo Fisher Waltham, Massachusetts), and incubated until an 80% confluence was reached. Later, the culture medium was removed and 100 μL of fresh medium, which contained C1 and NTBD (0–128 μg/mL) or AmB (0.125–0.5 μg/mL), were added. The cells were also exposed to combinations of AmB with the thiadiazoles. The individual mixtures contained C1 at concentrations of 8 μg/mL or 16 μg/mL and AmB at concentrations of 0.125 μg/mL or 0.25 μg/mL. In the combination with NTBD (4 or 8 μg/mL), AmB was used at a concentration of 0.25 or 0.5 μg/mL. After the 24/48-h treatment, an MTT solution (5 mg/mL; Sigma-Aldrich) was added to each well, and the cells were incubated for 3 h. Then, formazan crystals were solubilized overnight in SDS buffer (10% SDS in 0.01N HCl), and the product was quantified spectrophotometrically by measuring absorbance at the 570 nm wavelength using a microplate reader. Cell viability was calculated relative to the untreated (vehicle) control, whose viability was arbitrarily assumed to be 100%.

### 4.4. Flow Cytometry

Apoptotic and necrotic cells were examined using the flow cytometry technique, as described previously [16]. Briefly, 1 × 10^6^ cells/mL were seeded in 6-well plates and were allowed to attach overnight. The cells were then incubated for 24 h with selected concentrations of C1/NTBD and AmB or combinations of these drugs. Following the treatment, the cells were trypsinized, harvested, washed with PBS, and stained with 5 µL of AnnexinV/FITC and 5 µL of PI (50 µg/mL) (BD Biosciences, BD Pharmingen™, San Jose, CA, USA). After incubation for 15 min in the dark at room temperature, 5000 cells were immediately analyzed by flow cytometry (BD FACSCalibur) with CellQuest Pro Version 6.0 software (Becton Dickenson, San Jose, CA, USA). All experiments were performed in triplicate and yielded similar results.

### 4.5. Determination of ROS

The RPTECs were plated into 96-well black-walled plate (100 μL/well) and incubated at 37 °C in a humidified atmosphere with 5% CO_2_ to reach 80% confluency. The cells were then exposed to the thiadiazole derivatives (C1 and NTBD) and AmB alone or to the mixture of these compounds for 12 h. Next, the medium was removed, and a 20 μM solution of dihydrorhodamine 123 (DHR 12, Sigma-Aldrich) in PBS was added to each well (100 μL/well). DHR123 is a mitochondrial ROS indicator that can be used for the evaluation of mitochondrial oxidative stress. The cells were cultured for an additional 30 min at 37 °C in a humidified atmosphere with 5% CO_2_, washed three times with PBS, and the green fluorescence signal of the ROS-sensitive dye was measured in a fluorescence microplate reader using excitation at NTBD nm and emission at 525 nm.

### 4.6. Caspase Activity Assay

The caspase-2, -3 and -9 activity was determined in the cell lysates using a commercially available Caspase-2 Assay Kit (Colorimetric), Caspase-3 Assay Kit (Colorimetric), and Caspase-9 Assay Kit (Colorimetric) (Abcam, Cambridge, UK) according to the manufacturer’s instructions. Following the 24-h treatment of the RPTECs with the thiadiazole derivatives and AmB at the selected concentrations, the cells were trypsynized, washed with PBS, resuspended in a chilled cell lysis buffer, and incubated on ice for 10 min. After incubation, the cell lysates were centrifuged and the supernatants were used for the measurement of the protein concentration and the activity of caspases. 

The absorbance at the 405 nm wavelength was read on a BioTek Epoch Microplate Spectrophotometer (BioTek Instruments, Agilent Technologies, Santa Clara, CA, USA). The caspase activity was calculated relative to the cellular protein content, which was measured using a Pierce™ BCA Protein Assay Kit (Thermo Scientific, Waltham, MA, USA).

Each experiment was repeated at least three times. Statistical analyses were performed using GraphPAD Prism version 5 (GraphPAD Software Inc., San Diego, CA, USA) and Statistica 13.3 software (StatSoft, Tulsa, OK, USA). The data were analyzed by one-way ANOVA test, followed by Dunnett’s or Tukey’s multiple comparison tests. Values were expressed as means ± SD, and *p* values < 0.05 were considered significant.

### 4.7. Sample Preparation for ATR-FTIR Semi-Quantitative Analysis

Following the 24-h incubation of the RPTECs cultured in 6-well plates with or without the tested compounds (AmB1 0.125 µg/mL, AmB2 0.25 µg/mL, C1 8 µg/mL, NTBD 4 µg/mL, C1+AmB1, NTBD+AmB2), the media were discarded and the cells were washed with pre-cooled PBS and digested with trypsin. Next, the cells were resuspended in 0.9% NaCl and centrifuged at 1500 rpm for 5 min. Immediately after the addition of 4% paraformaldehyde to the cell pellet, the samples were stored at 4 °C overnight. Before the analysis, the cells were centrifuged and washed three times in 200 µL of 0.9% NaCl and twice in 200 µL of deionized water. The control samples and those subjected to the compounds were prepared in five replications.

### 4.8. ATR-FTIR Spectroscopy and Semi-Quantitative Data Analysis

For ATR-FTIR measurements, 60 µL of the final suspension of each sample was distributed on a ZnSe crystal and air dried. The ATR-FTIR spectra in the wave number range 4000–700 cm^−1^ were collected for all analyzed isolates using the FTIR VERTEX 70 spectrometer (Bruker Optic GmbH, Ettlingen, Germany) with an MCT detector. The IR spectra were sampled with a 2 cm^−1^ spectral resolution, and 64 scans were averaged per each sample spectrum. The baseline correction, vector normalization, calculation of reverse second derivatives as well as the calculation of the area of bands were performed using OPUS 7.5 software (Bruker Optic GmbH, Ettlingen, Germany). The Origin Pro 2020b program (OriginLab Corporation, Northampton, MA, USA) was used for the graphical processing of spectra and their reverse second derivatives. 

The statistical analysis of changes in the IR spectra of the RPTECs treated with C1, NTBD, AmB as well as C1+AmB and NTBD+AmB, compared to the controls, was performed with STATISTICA 7.1 software (StatSoft. Inc. 2005, Tulsa, OK, USA). To assess the statistical significance of the differences in the biomolecular content of the RPTEC, the non-parametric Mann–Whitney U test was applied; results with *p* < 0.05 were considered significant, and those with *p* < 0.1 were regarded as statistical trends. The choice of the non-parametric test was dictated by the fact that the analyzed data did not fulfill the assumptions of normality and homoscedasticity, which are necessary for the use of its parametric alternative.

## Figures and Tables

**Table 1:** Characterization of IR bands and band ratios analyzed in the ATR-FTIR study of RPTECs [31,36,43,47,48,49,50,51,52,53,54].

Band [cm^−1^] or Bands Ratio	Origin	Characteristics
3008	=CH str.	Level of unsaturated lipids
2958	CH_3_ str. asym.	Level of saturated lipids
2921	CH_2_ str. asym.	
2873	CH_3_ str. sym.	
2851	CH_2_ str. sym.	
1740	C=O str.	Level of lipids, phospholipids, esters
1687	C=O str., NH bend. (Amide I)	Level of proteins (antiparallel β-sheet and β-turn)
1654		Level of proteins (α-helix and Amide I maximum)
1635		Level of proteins (β-sheet)
1541	NH bend., CN str. (Amide II)	Level of proteins
1360–1480	CH_2_, CH_3_ def.	Level of proteins and lipids
1335	CN def. (Amide III)	Level of proteins
1310		
3008/2958	=CH str./CH_3_ str. asym.	Changes in lipid unsaturation level
2921/2958	CH_2_ str. asym./CH_3_ str. asym.	Changes in lipid chain length, branching, and/or saturation level
2851/2873	CH_2_ str. sym./CH_3_ str. sym.	
1635/1654	β-sheet/α-helix	Changes in relative level of β-sheet and α-helix secondary structures
1541/1654	Amide II/Amide I	Changes in secondary structure of proteins

## Data Availability

Data reported in this manuscript will be available upon request.
